# Jejunal Gastrointestinal Stromal Tumor Presenting as Life-Threatening Melena and Profound Anemia: A Case Report

**DOI:** 10.7759/cureus.110827

**Published:** 2026-06-14

**Authors:** Balakrishna Ravella, Eswar Chand Gadde, Harish Ravipati, Nestor Pamatmat

**Affiliations:** 1 Internal Medicine, OSF HealthCare St. Mary Medical Center, Galesburg, USA; 2 Internal Medicine, Ascension St. John Hospital, Tulsa, USA; 3 Internal Medicine, Guthrie Cortland Medical Center, Cortland, USA; 4 Pathology, OSF HealthCare St. Mary Medical Center, Galesburg, USA

**Keywords:** gastrointestinal stromal tumor, jejunal gist, melena, severe anemia, small bowel bleeding, surgical resection

## Abstract

Gastrointestinal stromal tumors (GISTs) are rare mesenchymal neoplasms and the most common sarcomas of the gastrointestinal tract. Small bowel GISTs, particularly jejunal tumors, frequently present with occult or overt bleeding leading to anemia, but are uncommon causes of melena when routine endoscopy has not been performed. We report a 67-year-old man who presented with one week of intermittent melena and profound anemia (hemoglobin 5.3 g/dL). Computed tomography (CT) angiography revealed a 5 cm jejunal mass without active bleeding. The patient underwent exploratory laparotomy with segmental resection and side-to-side anastomosis. Pathology confirmed a low-grade spindle cell GIST confined to the submucosa and muscularis propria, with strong diffuse CD117 and DOG1 positivity, scattered CD34 staining, and a Ki-67 index of ~3%. The patient recovered uneventfully with resolution of bleeding and normalization of hemoglobin. This case highlights the importance of considering small bowel GIST in unexplained melena and severe anemia and demonstrates the curative potential of timely surgical resection in localized low-risk disease.

## Introduction

Gastrointestinal stromal tumors (GISTs) are the most common mesenchymal neoplasms of the gastrointestinal tract, arising from the interstitial cells of Cajal [1]. Although they account for less than 1% of all gastrointestinal tumors, they represent the majority of sarcomas in this location [2]. The annual incidence in the United States is approximately 0.7 per 100,000 persons [3]. Most GISTs arise in the stomach (55-60%), whereas small intestinal GISTs comprise 25-35% of cases, with jejunal tumors representing a relatively uncommon subset [4].

Compared with gastric GISTs, small bowel lesions have a greater propensity for gastrointestinal bleeding because of their frequent submucosal or exophytic growth pattern, which predisposes to mucosal ulceration, melena, and iron-deficiency anemia. Because these tumors often lie beyond the reach of standard esophagogastroduodenoscopy (EGD) and colonoscopy, diagnosis may be delayed and frequently depends on cross-sectional imaging. Surgical resection remains the cornerstone of treatment for localized disease and offers excellent outcomes in low-risk tumors [5]. GISTs are commonly characterized by activating KIT or platelet-derived growth factor receptor A (PDGFRA) mutations and are confirmed immunohistochemically by strong positivity for CD117 and DOG1 [6]. This case of jejunal GIST presenting as life-threatening melena and severe anemia highlights the diagnostic challenges of small bowel neoplasms and underscores the importance of prompt imaging in patients with unexplained gastrointestinal bleeding.

## Case presentation

A 67-year-old man with a history of mixed hyperlipidemia, gastroesophageal reflux disease, and peripheral vascular disease status post right common iliac artery stent presented to the emergency department with one week of daily intermittent melena accompanied by lightheadedness, dizziness, and exertional dyspnea. The patient denied any prior episodes of melena, hematochezia, or hematemesis and had never undergone prior EGD or colonoscopy. He denied alcohol use, nonsteroidal anti-inflammatory drugs (NSAID) use, or recent medication changes (Table 1). The patient reported no significant family medical history, including no family history of malignancies.

**Table 1:** List of the patient's home medications at the time of admission

Medication	Dose
Aspirin	81 mg daily
Atorvastatin	20 mg daily
Clopidogrel	75 mg daily
Pantoprazole	40 mg once daily
Multivitamin	1 capsule daily
Cyanocobalamin	1000 mcg once daily

Vital signs on admission were temperature of 98.7 °F, heart rate of 100 beats/min, respiratory rate of 14/min, and blood pressure of 110/60 mm Hg. Physical examination revealed conjunctival pallor; the abdomen was soft, nontender, and without organomegaly. Laboratory evaluation was significant for profound anemia, with hemoglobin of 5.3 g/dL and low iron levels (Table 2). Prior to abdominal imaging, the leading diagnosis was acute gastrointestinal blood loss anemia secondary to upper gastrointestinal bleeding. Differential diagnoses included peptic ulcer disease, gastritis, esophagitis, angiodysplasia, small bowel arteriovenous malformations, and other causes of obscure gastrointestinal bleeding. CT angiography of the abdomen and pelvis revealed a 5 cm jejunal mass without active contrast extravasation (Figure 1). EGD was deferred because CT angiography had already identified a 5 cm jejunal mass requiring surgical evaluation and management. Additional endoscopic investigation was considered unlikely to alter the immediate treatment plan, and general surgery was therefore consulted. 

**Table 2:** Laboratory values on admission

Parameter	Value	Reference range
White blood cell count	5.35 × 10³/µL	4.5-11.0 × 10³/µL
Hemoglobin	5.3 g/dL	13.5-17.5 g/dL
Hematocrit	16.4%	41-50%
Mean corpuscular volume (MCV)	82 fL	80-100 fL
Platelet count	360 × 10³/µL	150-450 × 10³/µL
Sodium	138 mEq/L	135-145 mEq/L
Potassium	3.6 mEq/L	3.5-5.0 mEq/L
Blood urea nitrogen (BUN)	8 mg/dL	7-20 mg/dL
Creatinine	0.86 mg/dL	0.7-1.3 mg/dL
Aspartate aminotransferase (AST)	19 U/L	10-40 U/L
Alanine aminotransferase (ALT)	21 U/L	7-56 U/L
Alkaline phosphatase	35 U/L	44-147 U/L
Magnesium	2.0 mg/dL	1.7-2.2 mg/dL
Iron	15 µg/dL	50-150 µg/dL
Transferrin	228 mg/dL	200-360 mg/dL
Total iron binding capacity (TIBC)	285 µg/dL	250-450 µg/dL
Iron saturation	5%	15-50%

**Figure 1:**
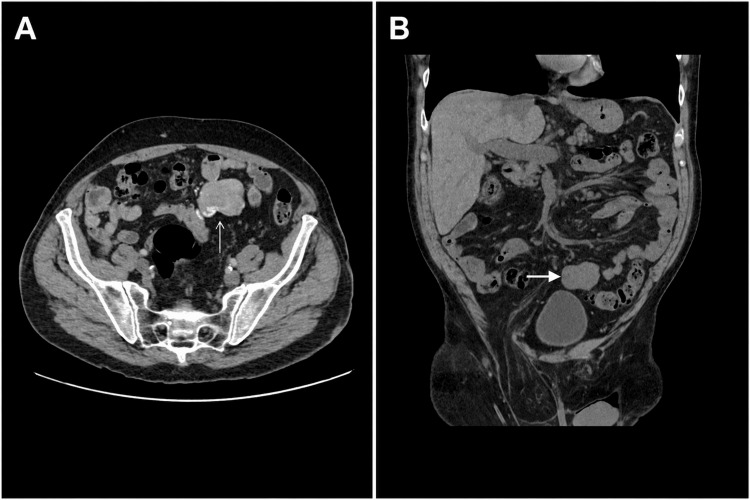
Jejunal mass (A) Axial view of computed tomography scan of the abdomen and pelvis showing the jejunal mass (white arrow). (B) Coronal view of the computed tomography scan of the abdomen and pelvis showing the jejunal mass (white arrow).

The patient received a total of four units of packed red blood cells, was made nil per os, and was started on 0.9% sodium chloride intravenous fluid hydration and intravenous pantoprazole 40 mg twice daily. His home medication of clopidogrel 75 mg daily was withheld upon admission to the hospital, given ongoing melena. The patient underwent laparoscopic-assisted segmental resection of the jejunal mass with primary side-to-side small bowel anastomosis. Intraoperatively, an approximately 5 cm localized jejunal mass was identified and successfully resected with grossly negative margins. No evidence of bowel perforation, adjacent organ invasion, or metastatic disease was observed.

His postoperative course was uncomplicated, with complete resolution of melena and stabilization of hemoglobin. The patient remained hospitalized for three days following surgery and was discharged home in stable condition. Given the low-risk pathological features, complete surgical resection with negative margins, and absence of metastatic disease, adjuvant imatinib therapy was not recommended.

Pathologic examination of the resected jejunal mass demonstrated a low-grade spindle cell GIST confined to the submucosa and muscularis propria (Figure 2). 

**Figure 2:**
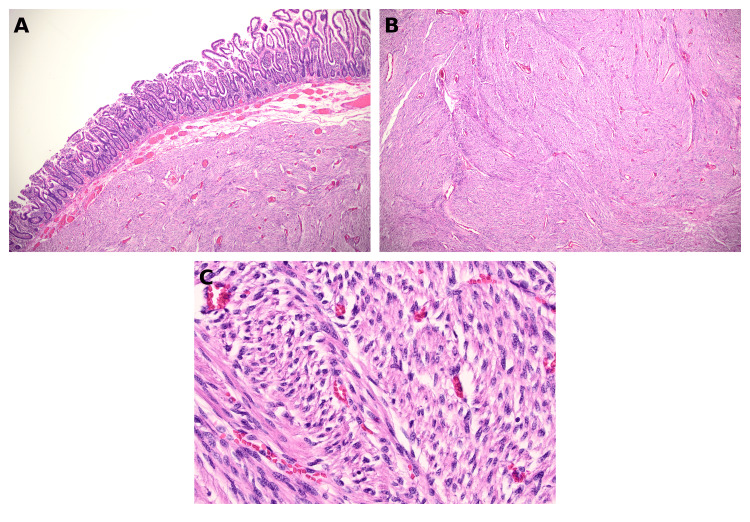
Histopathological examination (A) and (B) Low-power and high-power images of the standard hematoxylin and eosin slides. The lesion shows spindle cell morphology with fascicles of spindle cells with scattered nuclear atypia and ample eosinophilic cytoplasm that shows some skeinoid fibers. (C) A high-power magnification showing spindle cell morphology with fascicles of spindle cells.

Immunostains showed diffuse and strong positivity for CD117 and DOG1. CD34 showed scattered positivity throughout the tumor (Figure 3).

**Figure 3:**
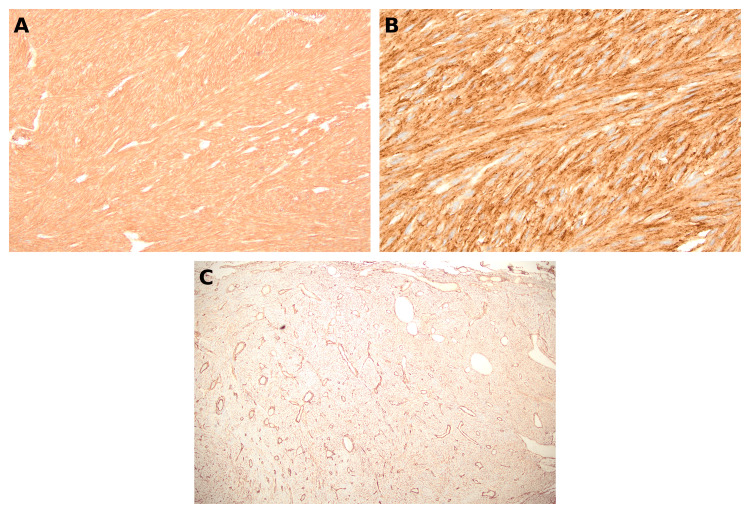
Diagnostic immunostains (A) CD117 immunostain diffuse positivity. (B) DOG1 immunostain diffuse positivity. (C) CD34 immunostain positivity.

Other immunostains, including pankeratin, S100, STAT6, and desmin, were negative in the tumor cells. Ki-67 immunostain was positive in only approximately 3% of tumor cells, consistent with the low-grade nature of the GIST. The mitotic rate was approximately 2 mitoses per 5 mm². The resected margins were negative. 

Two months after discharge, an outpatient colonoscopy identified four colonic polyps: two in the hepatic flexure, one in the transverse colon, and one in the descending colon, all of which were resected endoscopically and confirmed as tubular adenomas. Histologic images of the adenomas were not available for review. The patient subsequently followed up with hematology and oncology and is currently undergoing surveillance with CT imaging of the chest, abdomen, and pelvis every six months. At the 12-month follow-up, there was no clinical or radiographic evidence of disease recurrence, and his hemoglobin had normalized (Table 3). 

**Table 3:** Hemoglobin and hematocrit trends

Time point	Hemoglobin (g/dL)	Hematocrit (%)
Two months prior to admission	15.0	44.0
On admission	5.3	16.4
Post transfusion (four units packed red blood cells)	8.5	26.9
12-month follow-up	12.8	38.2

## Discussion

Although GISTs are the most common mesenchymal neoplasms of the gastrointestinal tract, jejunal tumors remain an uncommon but clinically important cause of obscure gastrointestinal bleeding and severe anemia [7]. Small bowel GISTs account for 25-35% of all GISTs, with the jejunum representing a substantial subset [8]. In contrast to gastric GISTs, small intestinal tumors have a higher propensity for gastrointestinal bleeding, likely due to their frequent submucosal or exophytic growth pattern within a narrower lumen, leading to mucosal ulceration, erosion, and chronic or acute hemorrhage [9]. Jejunal GISTs often remain clinically silent until significant bleeding occurs, as illustrated in this case, where the patient presented with one week of melena and hemoglobin of 5.3 g/dL without obstructive symptoms or a palpable mass. The patient’s dual antiplatelet therapy with aspirin and clopidogrel likely contributed to the severity of bleeding from the ulcerated submucosal mass.

The diagnostic challenge in small bowel GISTs stems from their location beyond the reach of routine EGD and colonoscopy. Standard endoscopic evaluation is frequently nondiagnostic [10]. This underscores the critical role of cross-sectional imaging, particularly contrast-enhanced CT angiography of the abdomen and pelvis, which is highly sensitive for detecting the characteristic enhancing mass [11], assessing size, location, and potential bleeding, as demonstrated in this patient. In this case, the rapid identification of a 5 cm jejunal mass on CT angiography allowed prompt surgical consultation and intervention, preventing further hemodynamic compromise. Because cross-sectional imaging had already identified a surgically resectable jejunal mass, upper endoscopy was deferred, as it was unlikely to identify the culprit lesion or alter immediate management. Additional small bowel investigations, such as capsule endoscopy or device-assisted enteroscopy, were similarly not pursued because definitive surgical treatment had already been established.

Histologically, the majority of GISTs are the spindle cell type, as seen here. Immunohistochemistry is essential for diagnosis and differentiation from other mesenchymal tumors. Greater than 95% of GISTs demonstrate strong, diffuse positivity for CD117 (KIT), with DOG1 (ANO1) offering nearly equivalent or superior sensitivity [12]. Scattered CD34 positivity is common (approximately 70%), while negativity for epithelial (pankeratin), neural (S100), and smooth muscle (desmin) markers, along with STAT6 negativity, reliably excludes leiomyosarcoma, schwannoma, and solitary fibrous tumor [13]. The low Ki-67 proliferation index (approximately 3% in this case) correlates with low mitotic activity and supports low-grade biology [14].

Risk stratification for recurrence in GIST integrates tumor size, mitotic rate (per 50 high-power fields), anatomic location, and rupture status, as outlined in the modified National Institutes of Health (NIH) criteria and National Comprehensive Cancer Network (NCCN) guidelines [15]. A 5 cm jejunal GIST with a mitotic rate of approximately 2 mitoses per 5 mm² and a Ki-67 proliferation index of approximately 3% falls within the low-risk category for recurrence according to established risk stratification systems that incorporate tumor size, mitotic activity, anatomic location, and tumor rupture status. In the present case, the absence of tumor rupture, achievement of negative margins, and low proliferative activity supported a favorable prognosis.

Surgical resection with negative margins and preservation of adjacent structures remains the definitive treatment for localized, resectable GISTs and is curative in the majority of low-risk cases [16]. Laparoscopic or open segmental resection, as performed here via exploratory laparotomy with side-to-side anastomosis, is the standard approach. Adjuvant imatinib mesylate is not routinely recommended for low- or low-intermediate-risk tumors [15]. Long-term surveillance with CT imaging of the chest, abdomen, and pelvis every six months for the first five years (then annually) is recommended to monitor for recurrence, as was appropriately initiated in this patient. At 12-month follow-up, there was no evidence of disease recurrence, and hemoglobin had normalized to 12.8 g/dL.

This case adds to the growing but still limited literature on jejunal GIST presenting as isolated severe anemia from melena without prior endoscopic evaluation or other overt symptoms. It reinforces the need for a high index of suspicion for small bowel neoplasms in patients with unexplained gastrointestinal bleeding and profound anemia, particularly when routine endoscopy is unrevealing. Prompt cross-sectional imaging and multidisciplinary management (involving surgery, pathology, and oncology) are essential to achieve timely diagnosis and excellent outcomes in low-grade, localized disease.

## Conclusions

This case illustrates a jejunal GIST presenting with life-threatening melena and severe anemia (hemoglobin 5.3 g/dL) that was successfully managed with prompt surgical resection. Although small bowel GISTs are uncommon, they should be considered in the differential diagnosis of obscure gastrointestinal bleeding when standard endoscopy is negative or deferred. Early cross-sectional imaging, timely surgical intervention, and appropriate follow-up can lead to excellent outcomes in low-risk, localized disease. Clinicians should maintain a high index of suspicion for small bowel neoplasms in patients with unexplained melena and profound anemia to prevent life-threatening hemorrhage and enable curative treatment.

## References

[1] Parab TM, DeRogatis MJ, Boaz AM (2019). Gastrointestinal stromal tumors: a comprehensive review. J Gastrointest Oncol.

[2] Alvarez CS, Piazuelo MB, Fleitas-Kanonnikoff T, Ruhl J, Pérez-Fidalgo JA, Camargo MC (2024). Incidence and survival outcomes of gastrointestinal stromal tumors. JAMA Netw Open.

[3] Zhou L, Liao Y, Wu J, Yang J, Zhang H, Wang X, Sun S (2018). Small bowel gastrointestinal stromal tumor: a retrospective study of 32 cases at a single center and review of the literature. Ther Clin Risk Manag.

[4] Huynh AT, Rust A (2024). Jejunal gastrointestinal stromal tumor (GIST) with profound anemia. J Surg Case Rep.

[5] von Mehren M, Kane JM, Riedel RF (2022). NCCN Guidelines® insights: gastrointestinal stromal tumors, version 2.2022. J Natl Compr Canc Netw.

[6] Espinosa I, Lee CH, Kim MK (2008). A novel monoclonal antibody against DOG1 is a sensitive and specific marker for gastrointestinal stromal tumors. Am J Surg Pathol.

[7] Mandalà S, Lupo M, Guccione M, La Barbera C, Iadicola D, Mirabella A (2021). Small bowel gastrointestinal stromal tumor presenting with gastrointestinal bleeding in patient with type 1 neurofibromatosis: management and laparoscopic treatment. Case report and review of the literature. Int J Surg Case Rep.

[8] Köseoğlu H, Solakoglu T, Akin F (2018). Intestinal gastrointestinal stromal tumors presenting with gastrointestinal bleeding. Eur J Gen Med.

[9] Mulkerrin G, Hogan NM, Sheehan M, Joyce MR (2018). Melena as an unusual presentation of gastrointestinal stromal tumour, a case report. Int J Surg Case Rep.

[10] Neppala S, Caravella J, Chigurupati H (2021). Jejunal gastrointestinal stromal tumor: a case report presenting with recurrent episodes of melena. Am J Gastroenterol.

[11] Choi HI, Choi MJ, Kim BM, Namgung H, Choi SK (2022). Identifying small bowel gastrointestinal stromal tumor as the culprit lesion in obscure gastrointestinal bleeding: emphasis on angiographic findings. Taehan Yongsang Uihakhoe Chi.

[12] Sözütek D, Yanık S, Akkoca AN (2014). Diagnostic and prognostic roles of DOG1 and Ki-67, in GIST patients with localized or advanced/metastatic disease. Int J Clin Exp Med.

[13] Ríos-Moreno MJ, Jaramillo S, Pereira Gallardo S (2012). Gastrointestinal stromal tumors (GISTs): CD117, DOG-1 and PKCθ expression. Is there any advantage in using several markers?. Pathol Res Pract.

[14] Kadado KJ, Abernathy OL, Salyers WJ, Kallail KJ (2022). Gastrointestinal stromal tumor and Ki-67 as a prognostic indicator. Cureus.

[15] National Comprehensive Cancer Network. Gastrointestinal Stromal Tumors (GIST) Version 1.2026. https://www.nccn.org/guidelines/guidelines-detail?category=1&id=1507.

[16] Azimi B, Shahrbaf MA, Iranshahi M, Parsaeian F (2022). A case of jejunal GIST revealed by hematemesis: unusual situation. Int J Surg Case Rep.

